# Is Supplemental Oxygen Needed in Cardiac Compression?—The Influence of Oxygen on Cerebral Perfusion in Severely Asphyxiated Neonates With Bradycardia or Cardiac Asystole

**DOI:** 10.3389/fped.2019.00486

**Published:** 2019-11-20

**Authors:** Anne Lee Solevåg, Georg M. Schmölzer, Po-Yin Cheung

**Affiliations:** ^1^Department of Paediatric and Adolescent Medicine, Akershus University Hospital, Lørenskog, Norway; ^2^Neonatal Research Unit, Centre for the Studies of Asphyxia and Resuscitation, Royal Alexandra Hospital, Edmonton, AB, Canada; ^3^Department of Pediatrics, University of Alberta, Edmonton, AB, Canada

**Keywords:** newborn infant, asphyxia, oxygen, chest compression, cerebral perfusion

## Abstract

**Background:** Previous studies have investigated hemodynamic recovery using 21% vs. 100% oxygen during cardiopulmonary resuscitation (CPR) with chest compression (CC) in term infants. Animal studies indicate that systemic circulatory recovery is the same whether 21 or 100% oxygen is used during neonatal CPR. One of the main goals of resuscitation is to maintain cerebral oxygen delivery and prevent cerebral hypo- and hyperoxygenation. Oxygen delivery to the brain depends on cerebral hemodynamics, concentration of inhaled oxygen and blood oxygen content. The aim of this paper was to synthesize available research about cerebral oxygen delivery during CPR using different oxygen concentrations. Our research questions included how do different oxygen concentrations during CPR with CC influence cerebral perfusion and oxygen delivery, and how do cerebral hemodynamics during CC influence outcomes.

**Methods:** A search in Medline Ovid using the search terms hypoxia AND oxygen AND cerebrovascular circulation AND infant, newborn. Inclusion criteria included studies of hypoxia and resuscitation of term infants. Studies were excluded if no measures of cerebral blood flow (CBF), oxygenation, or perfusion were reported.

**Results:** The search retrieved 21 papers. None of the studies directly fulfilled our inclusion criteria. The reference lists of some of the retrieved papers provided relevant animal studies with slightly conflicting results regarding blood flow and oxygen delivery to the brain using 21 or 100% oxygen. No study in term infants was identified, but we included one study in preterm infants. Studies in asphyxiated animals indicate that 100% oxygen increases CBF and oxygenation during and after CC with a potential increase in oxidative stress.

**Conclusion:** In asphyxia, cerebral autoregulation may be impaired. Pure oxygen administration during CC may result in cerebral hyperperfusion and increased cerebral oxygen delivery, which may be associated with oxidative stress-related damage to the brain tissue. As systemic circulatory recovery is the same whether 21 or 100% oxygen is used during neonatal CPR, it is important to investigate whether brain damage could be aggravated when 100% oxygen is used.

## Introduction

Despite a lack of scientific evidence, supplemental oxygen has been used in neonatal resuscitation for more than 200 years (1). For the last few decades, research has been focused on the balancing benefits and potential harms associated with different oxygen concentrations used in delivery room resuscitation. In term infants exposed to a high initial oxygen concentration in the delivery room, neonatal mortality is increased (2). Since 2010, an initial oxygen concentration of 21% has been recommended to term infants that require assisted ventilation, but 100% oxygen is recommended as soon as chest compression (CC) is needed (3). Exposure to oxygen during resuscitation can be limited in two ways, either by reducing the fraction of inspired oxygen (FiO_2_) or by limiting the time of exposure (4). In term infants, the oxygen concentration has been the focus of interest, although some animal studies have addressed the effect of very brief (e.g., 3 min) or limited exposure to 100% oxygen (5–7). No study has assessed the safety and effect of different oxygen exposures after return of spontaneous circulation (ROSC) in term asphyxiated infants, i.e., cumulative oxygen exposure. In addition, long-term data applicable to term infants after CC are lacking. Although data from the Canadian Neonatal Network indicated an increased risk of neurodevelopmental impairment in premature infants <29 weeks resuscitated with 100% oxygen (8), a meta-analysis of eight studies showed no difference in the risk of bronchopulmonary dysplasia, intraventricular hemorrhage >grade 3, or death in premature infants <29 weeks of gestation receiving higher vs. lower oxygen strategies in the delivery room (9). In premature infants <32 weeks of gestation, Oei et al. (10) aimed to examine disability at 2 years after initial delivery room exposure to 21 or 100% oxygen. FiO_2_ was adjusted to target SpO_2_ 65–95% at 5 min and 85–95% until admission to the neonatal intensive care unit. At 2 years of age, 215 out of 240 survivors were assessed (11). There was no difference in disability between infants initially receiving 21% vs. 100% oxygen (11). This was in agreement with a meta-analysis of two trials (*n* = 208) that showed no difference in neurodevelopmental disability at 18–24 months between premature infants (<32 weeks and extremely low birth weight, respectively) receiving lower (FiO_2_ < 0.4) vs. higher (FiO_2_ > 0.4) initial oxygen concentrations targeted to oxygen saturation (12).

One of the main goals of resuscitation is to maintain cerebral oxygen delivery and prevent cerebral hypo- and hyperoxygenation (13). Cerebral oxygen delivery is determined by cerebral hemodynamics, concentration of inhaled oxygen, and blood oxygen content as determined by pulmonary gas exchange and hemoglobin concentration (14). Cerebral hemodynamic measurements include cerebral blood flow (CBF) and cerebral blood flow velocity (CBFV). Both hypoxemia and hyperoxemia influence CBF (15). A review (16) and a meta-analysis (17) have concluded that 21% oxygen is equivalent to 100% oxygen regarding the rate of and time to ROSC, as well as post resuscitation mortality and morbidity in asphyxiated animals The aim of this paper is to provide an overview of studies that assessed CBF, directly or indirectly, during cardiopulmonary resuscitation (CPR) including CC with different FiO_2_. Our research questions were how did FiO_2_ during CPR with CC influence cerebral perfusion and oxygenation, and how did cerebral hemodynamics during CC influence outcomes. We hypothesized that cerebral autoregulation was impaired in infants that required delivery room CPR with CC, and that high cerebral perfusion and oxygen delivery was not beneficial.

## Materials and Methods

A search in Medline Ovid was performed in July 2019 including the search terms hypoxia AND oxygen AND cerebrovascular circulation AND infant, newborn. Conference proceedings and the reference list of retrieved papers were hand searched for relevant researchers and papers. Publications were assessed based on title, abstract, and methods. Studies were included if they addressed hypoxia and resuscitation in term infants. Studies were excluded if no measures of CBF/perfusion and/or oxygenation were reported, or if they were in a different language than English or Scandinavian.

## Results

### Results of the Literature Search

(1) The Medline Ovid search retrieved 21 papers that were all excluded: 7 because they addressed premature infants (18–24). One paper in Japanese (25) and one in German (26) was excluded, and 12 papers did not address resuscitation (27–38). From the reference list of (21), we identified (4), which further identified (39).

(2) Papers that we identified from hand searches of conference proceedings and reference lists: there were no clinical data evaluating cerebral hemodynamics and oxygenation during CC in newborn term infants, but four animal studies were identified where CBF/perfusion and/or oxygenation were reported during CC (Table 1 presents CBF/perfusion/oxygen data).

**Table 1:** Study design, subjects, and main findings related to cerebral oxygenation/perfusion.

	**Design**	**Reported according to ARRIVE**	**Subjects**	**Age**	**Weight**	**ROSC definition**	**Time to ROSC**	**Post-ROSC observation**	**Method for assessing cerebral oxygenation/perfusion**	**Main results**
Rawat et al. (40)	Secondary analysis of three previous studies (not cited)	No power calculation Not randomized	Lamb	Fetal	3.7 kg	HR > 60/min with a SBP > 30 mm Hg	Mean ± standard deviation 21% O_2_ 211 ± 145 s 100% O_2_ 306 ± 270 s	30 min	Flow probe around the left CA—not specified internal or external CA Oxygen delivery was calculated as oxygen content in the carotid arterial blood (CaO_2_) × carotid blood flow where CaO_2_ = hemoglobin × 1.34 × SaO_2_/100 + 0.0031 × PaO_2_ (in mmHg)	During CC, PaO_2_ was not different between 21 and 100% oxygen ventilated lambs, but CA flow was 1.2 (1.6) mL/kg/min (21% oxygen) vs. 3.2 (3.4) mL/kg/min (100% oxygen); *p* = 0.07 Oxygen delivery to the brain was 0.05 (0.06) mL/kg/min (21% oxygen) vs. 0.11 (0.09) mL/kg/min (100% oxygen); *p* < 0.001. Immediately after ROSC, lambs ventilated with 100% oxygen had higher PaO_2_ and pulmonary blood flow.
Perez-de-Sa et al. (5)	Randomized controlled animal trial	No power calculation	Lamb	Fetal	3.4 kg	HR > 150/min	Median (interquartile range) 21% O_2_ 68 (6–150) s 100% O_2_ 3 min 107 (5–182) s 100% O_2_ 30 min 58 (23–368) s	60 min	Partial pressure of oxygen in brain tissue (PbtO_2_) Near infrared spectroscopy regional cerebral saturation (CrSO_2_)	Limiting the time of exposure to 100% oxygen to 3 min did not avoid brain tissue hyperoxia.
Linner et al. (6)	Randomized controlled animal trial	No power calculation Randomization, but randomization procedure not explained	“Domestic” piglets	12–36 h	1.4–1.8 kg	MAP > 40 mmHg and HR > 150/min	Median (interquartile range) 21% O_2_ 67 (60–76) s 100% O_2_ 3 min 88 (76–126) s 100% O_2_ 30 min 68 (56–81) s	4 h	Partial pressure of oxygen in brain tissue (PbtO_2_) Near infrared spectroscopy regional cerebral saturation (CrSO_2_)	Maximum PbtO_2_ was 12 kPa (90 mmHg) (6.4 kPa (48 mmHg)) −15 kPa (112 mmHg) and 25 kPa (187 mmHg) (15 kPa (112 mmHg)) −36 kPa (270 mmHg) in piglets exposed to 100% oxygen for 3 and 30 min, respectively. In the 21% oxygen ventilated piglets, maximum PbtO_2_ was 4.2 kPa (31 mmHg) (3.3 kPa (25 mmHg)) −5.4 kPa (40 mmHg).
Linner et al. (7)	Randomized controlled animal trial	Power calculation performed Randomization and blinding, but randomization procedure not explained	“Domestic” piglets	12–36 h	1.4–1.8 kg	MAP > 40 mmHg and HR > 150/min	Median (interquartile range) 21% O_2_ 67 (60–76) s 100% O_2_ 3 min 88 (76–126) s 100% O_2_ 30 min 68 (56–81) s	4 h	Partial pressure of oxygen in brain tissue (PbtO_2_) Near infrared spectroscopy regional cerebral saturation (CrSO_2_)	CrSO_2_-values were higher in the piglets that received one inflation with 100% oxygen per minute, but no brain hyperoxia was demonstrated: Maximum PaO_2_ during inadequate ventilation with 100% oxygen was 9.6 kPa (72 mmHg), and the highest PbtO_2_ was 5.7 kPa (43 mmHg).
Presti et al. (41)	Controlled animal trial	Power calculation not performed Non-randomized	Mice	3 days	Not stated	N/A Not arrest	N/A	24 h and 7 weeks	13 mice were used to study CBF: Laser doppler flowmetry. The changes in CBF were recorded before, during, and 10 min after hypoxia and expressed as a percentage of the prehypoxic level.	CBF was re-established faster with 100% (*n* = 6) vs. 21% oxygen (*n* = 7) in neonatal and adult mice. However, 100% oxygen resulted in cerebral hyperperfusion (150% of prehypoxic levels). The same pattern was not seen in mice exposed to 21% oxygen.
Solas et al. (39)	Randomized controlled animal trial	No power calculation Randomization, but randomization procedure not explained	Noroc piglets	1–3 days	1.4–2.7 kg	N/A Not arrest model	N/A	2 h	Laser Doppler flowmetry	MAP was lower and cerebral cortical hypoperfusion more pronounced in in piglets exposed to 21% oxygen vs. 100% oxygen.
Solas et al. (4)	Randomized controlled animal trial	No power calculation Randomization, but randomization procedure not explained	Noroc piglets	1–3 days	1.1–2.5 kg	N/A Not arrest model	N/A	2 h	Laser Doppler flowmetry	There was a higher oxygen delivery in the two groups (5 or 20 min) receiving 100% oxygen and a close to significantly higher oxygen extraction ratio in the 21% oxygen group.
Solas et al. (42)	Randomized controlled animal trial	No power calculation Randomization, but randomization procedure not explained	Noroc piglets	1–3 days	1.0–2.1 kg	N/A Not arrest model	N/A	2 h	Laser Doppler flowmetry	PaO_2_ during the first 30 min of reoxygenation-reperfusion was higher in piglets ventilated with 100% oxygen. MAP was also higher compared with piglets ventilated with 21% oxygen. MAP reached baseline values after 10 min of reoxygenation-reperfusion in the 100% oxygen group and after 30 min in the 21% oxygen group. Cerebral microcirculation was re-established faster in the group receiving 100% oxygen vs. the 21% oxygen group. Repeated-measures ANOVA for the whole reoxygenation- reperfusion period showed an ~20% higher CBF in the 100% oxygen group.
Richards et al. (43)	Randomized controlled animal trial	No power calculation Block randomization, but randomization procedure nor explained	Yorkshire-Landrace piglets	1–3 days	1.5–2.1 kg	N/A Not arrest model: PaO_2_ 20–40 mmHg for 2 h	N/A	4 h	Left common CA flow Carotid vascular resistance was calculated as (mean arterial pressure–central venous pressure)/flow in the common CA	Following reoxygenation CBF was higher in piglets resuscitated with 21% oxygen vs. both 50% and 100% oxygenCBF was not different between groups after 30 min of reoxygenation. There was a significant correlation between arterial partial pressure of oxygen and carotid vascular resistance
Soberg et al. (44)	Randomized controlled animal trial	No power calculation Block randomization, but randomization procedure not explained	Noroc piglets	14–36 h	2.0–2.7 kg	N/A Not arrest model: MAP 15 mmH or BE −20 mmol/L	N/A	9 h	N/A	Piglets ventilated with 21% oxygen had significantly lower levels of neurofurans and neuroprostanes than piglets that were ventilated with supplementary oxygen in a dose-dependent manner, i.e., 21% <40% <100% oxygen. Isoprostanes were significantly lower in piglets ventilated with 40% vs. 100% oxygen.
Lundstrom et al. (45)	Randomized controlled trial	Power calculation performed to detect a 10% difference in CBF Randomization procedure not explained	Premature infants <33 weeks	Delivery room	550–2,590 g	N/A Not arrest	N/A	2 h	Xenon clearance	2 h after birth, CBF was 15.9 (13.6–21.9) ml/100 g/min in the infants that had been ventilated with 21% oxygen vs. 12.3 (10.7–13.8) ml/100 g/min in infants ventilated with 80% oxygen (*p* < 0.00001).

*Studies were identified through hand searches of conference proceedings and reference lists. CC, chest compression; ROSC, return of spontaneous circulation; HR, heart rate; MAP, mean arterial blood pressure; BE, base excess; SBP, systolic blood pressure; CA, carotid artery; CBF, cerebral blood flow; ARRIVE, Animal Research: Reporting in vivo Experiments*.

Table 1 gives an overview of the studies identified through hand searches with regards to study design, subjects and main findings related to cerebral oxygenation/perfusion and oxygenation.

Rawat et al. (40) performed secondary analyses of data from transitional lambs with cardiac arrest induced by umbilical cord occlusion (PaCO_2_ 125 mmHg = 16.7 kPa). After 5 min of asystole, ventilation was initiated with 21% oxygen. When CC was started, oxygen was increased to 100% (*n* = 25) or kept at 21% (*n* = 9). Lambs were observed for 30 min after ROSC during which FiO_2_ was titrated to a preductal SpO_2_ 85–95%. All lambs achieved ROSC after the same number of epinephrine doses. The authors concluded that carotid artery (CA) flow, systemic PaO_2_, and oxygen delivery to the brain were very low during CC irrespective of ventilation with 21 or 100% oxygen during CPR.

From the reference list of Rawat et al. (40), we identified a randomized trial by Perez-de-Sa et al. (5). They examined asphyxiated (PaCO_2_ 17 kPa = 127 mmHg) transitional lambs; CC was performed with 21% oxygen (*n* = 7) or 100% oxygen for 3 min (*n* = 6) or 30 min (*n* = 6). Systemic circulatory recovery measured by heart rate and blood pressure was similar in the three groups. One lamb randomized to resuscitation with oxygen for 30 min was excluded because of anemia. The remaining lambs survived to completion of the protocol. Messenger ribonucleic acid expression in the brain of interleukin (IL)-1ß, IL-12, and IL-18 were not different between the groups.

Also, from the reference list of Rawat et al. (40), we identified a randomized trial by Linner et al. (6) who used asphyxiated (PaCO_2_ 21 kPa = 157 mmHg) piglets to investigate if ventilation with 100% oxygen [for 3 min (*n* = 12) + for 30 min (*n* = 13)], instead of 21% oxygen (*n* = 13) would improve ROSC during CC. One piglet resuscitated with 21% oxygen did not achieve ROSC, whereas one piglet that was assigned to ventilation with oxygen for 30 min achieved ROSC, but died 10 min after initiation of ventilation. The time to recovery of cerebral oxygenation was defined as the time when CrSO_2_ reached 30%, and when PbtO_2_ had increased 0.1 kPa (0.75 mmHg) from its lowest level. They concluded that shortening the time of exposure to 100% oxygen to 3 min did not prevent brain tissue hyperoxia.

In a randomized controlled trial, Linner et al. (7) used the same piglet model to investigate whether one inflation per minute with 100% oxygen (*n* = 8) would improve recovery compared to one inflation per minute with 21% oxygen (*n* = 8). The setup was designed to mimic a situation with severely inadequate ventilation. At the end of 10 min of inadequate ventilation, the 21% oxygen group had a higher lactate and lower PaCO_2_ than the oxygen group. Two animals in the 21% oxygen group did not achieve ROSC.

(3) Several studies where no CC was provided were included to supplement the scarce data available from CC animal studies (Table 1).

### Supplemental Oxygen Increases CBF

Presti et al. (41) investigated CA ligated neonatal and adult mice that had been exposed to 20 min of 8% oxygen, followed by 30 min of 21 or 100% oxygen. PaCO_2_ values were not reported, but presumably, this was a model of normocapnia.

Laser Doppler flowmetry revealed a CBF of 150% of prehypoxic levels in mice exposed to 100% oxygen. Neonatal mice exposed to 100% oxygen (*n* = 32) had significantly delayed geotaxis reflex at 24 h, when compared with neonatal mice exposed to 21% oxygen (*n* = 37). In contrast, adult mice exposed to 100% oxygen (*n* = 18) demonstrated significantly better spatial learning and orientation with a tendency toward better memory preservation vs. mice exposed to 21% oxygen (*n* = 27). There was a trend toward a higher mortality among 100% resuscitated mice vs. those resuscitated with 21% oxygen.

In a randomized controlled design, Solas et al. (39) used a piglet model of cerebral hypoxemia-ischemia (8% oxygen and bilateral common CA occlusion) and demonstrated a significantly higher mean arterial blood pressure (MAP) and faster re-establishment of cerebral cortical microcirculation during ventilation with 100% oxygen for 30 min (*n* = 12) compared with 21% oxygen (*n* = 12) (39). Excitatory amino acids in the striatum were higher in the 21% oxygen group. No pig died in either group.

The investigators subsequently added moderate hypercapnia (mean PaCO_2_ 8.4 kPa = 63 mmHg) to the model and confirmed that cerebral cortical microcirculation was higher when piglets were ventilated with 100% oxygen for 5 min (*n* = 12) or 20 min (*n* = 12) compared with 21% oxygen (*n* = 12) (4). There were no differences in biochemical markers including excitatory amino acids in the striatum between the groups. No pig died in either group.

In a third randomized study by Solas et al. (42), piglets with moderate hypercapnia to more closely simulate perinatal asphyxia, were subjected to 20 min of combined hypoxemia-ischemia-hypercapnia followed by reperfusion and reoxygenation: (1) with 100% oxygen for the first 30 min and then 21% oxygen for another 90 min (*n* = 11), or (2) with 21% oxygen for 120 min (*n* = 13). No differences in biochemical markers were found between the two groups. No pig died in either group.

### Supplemental Oxygen Reduces CBF

In a randomized controlled trial of normocapnic hypoxemia, Richards et al. (43) subjected piglets to FiO_2_ 0.10–0.15 for 2 h and randomized them to reoxygenation with 21% oxygen (*n* = 8), 50% oxygen (*n* = 8), or 100% oxygen (*n* = 8) for 1 h, followed by 21% oxygen for 3 h. They found an oxygen dose-dependent increase in global matrix metalloproteinase-2 (MMP-2) activity in the brain. Based on the relationship between FiO_2_, CA vascular resistance and cerebral MMP-2 activity, the authors speculated that a higher PaO_2_ may play a role in vasoregulation through an oxidative stress related activation of peroxynitrite and vascular MMP-2.

Solberg et al. (44) conducted a randomized controlled trial in asphyxiated piglets (PaCO_2_ 8.6–8.9 kPa = 64–67 mmHg) to measure the levels of oxidative stress markers (lipid peroxidation-oxidation products of arachidonic acid and docosahexanoic acid) in the cerebral cortex after hypoxia and reoxygenation with 21% (*n* = 9), 40% (*n* = 12) or 100% oxygen (*n* = 8). Solberg et al. (44) did not measure CBF of perfusion, but discussed the fact that isoprostanes have been reported to be potent vasoconstrictors of brain vasculature (46). High values of isoprostanes measured in the 100% group could thereby promote cerebral vasoconstriction.

Lundstrom et al. (45) used Xenon clearance to measure CBF in premature infants <33 completed weeks of gestation. CBF was lower 2 h after randomization to <10 min ventilation with 80% oxygen (*n* = 35) compared to <10 min ventilation with 21% oxygen (*n* = 34) at birth. Median PaCO_2_ was 6 kPa (45 mmHg) (not different between the groups) at the time of measurement. The authors suggested a prolonged effect of hyperoxaemia, possibly mediated by an effect of toxic oxygen metabolites on the cerebral vasculature in premature infants. Cardiac left ventricular output was not significantly lower in the infants treated with 80% oxygen.

## Discussion

A fine balance exists between relaxing and contracting factors in vascular endothelial cells during asphyxia and reoxygenation-reperfusion, and oxygen radicals are potent regulators of cerebral arteriole and artery tone (47, 48). The results of this literature review were slightly conflicting regarding how FiO_2_ influences cerebral perfusion and oxygenation in neonatal animals and premature infants. Epinephrine and anesthetics may influence cerebral perfusion and -autoregulation, which may explain some of the differences in experimental animal studies. Epinephrine increases cerebral perfusion pressure (49), CBF (50, 51), and cerebral oxygen uptake (51). Solberg et al. (44) used isoflurane for induction in piglets. Isoflurane abolishes CBF autoregulation (52), but has a short half-life and was only used in the initial phases of the experiment.

Moderate hypercapnia may protect the brain from hypoxic-ischemic injury (39, 53). The mechanisms include reduced cerebral energy utilization (54) and preserved high-energy phosphate reserves (55). Hypercapnia also shifts the oxygen-hemoglobin dissociation curve to the right, resulting in increased oxygen unloading to the tissues despite tissue hypoperfusion. Solas et al. (42) demonstrated that MAP and microcirculation in the cerebral cortex decreased somewhat less during hypoxia-ischemia, and recovered more rapidly during reoxygenation-reperfusion when CO_2_ was added to the model. Although a higher MAP and cortical microcirculation was found in the 100% compared with the 21% group, the difference between the groups was less marked than in normocapnia (39). CO_2_ influences CBF, and both term animals and humans have a strong cerebrovascular sensitivity to changing PaCO_2_ (56–58), with an increase in CBF of 25%/kPa PaCO_2_ in healthy term babies. However, this CO_2_ reactivity may be lost in severe asphyxia (57).

Richards et al. (43) and Solberg et al. (44) speculated that elevated MMP-2 and isoprostanes, respectively, in piglets resuscitated with supplemental oxygen could promote cerebral vasoconstriction and thus a lower CBF. Based on other investigations reported in this paper, it is perhaps more likely that 100% oxygen, when administered after asphyxia, increases CBF. However, the results of Richards et al. (43) and Solberg et al. (44) are in agreement with studies that indicate that 100% oxygen exacerbates reperfusion injury and reduces cerebral perfusion in premature infants (45).

Autoregulation ensures cerebral perfusion and oxygenation by maintaining CBF if cerebral perfusion pressure changes (59). Under normal conditions, hyperoxia induces cerebral vasoconstriction (60), but this oxygen reactivity may be lost during tissue ischemia (61). Hyperoxia may work directly on vascular tone, but also indirectly through the formation of reactive oxygen species (62). The effects of hydrogen peroxide (H_2_O_2_) on vascular tone have been the most extensively studied. Exogenous H_2_O_2_ produces relaxation of cerebral arteries *in vitro* (48, 63–65).

Neonatal oxygen requirements and responsiveness of the cerebral vasculature to hyperoxia may differ at different levels of maturity and depend on perinatal factors. Fetal oxygen saturation is about 50%, and a healthy newborn requires at least 5 min to achieve an oxygen saturation >90% (66). The increase in oxygenation is delayed in infants with halted pulmonary vasodilatation, e.g., in chorioamnionitis where inflammation and pulmonary remodeling predispose to impaired gas exchange and persistent pulmonary hypertension. Initiation of breathing, together with the use of oxygen and mechanical ventilation contribute to oxidative stress and inflammation (67, 68), not only in the lungs, but systemically with consequences for other organs including the brain. In addition, altered pulmonary venous return and, subsequently, left ventricular output, result in CBF fluctuations. Supplemental oxygen contributes to decreased pulmonary vascular resistance and increased PBF and may thus be needed in e.g., infants born to mothers with chorioamnionitis. The effect of arterial oxygen tension on pulmonary arterial pressure and ductal shunting is gestational age dependent. Left to right ductal shunting may result in cerebral hypoperfusion secondary to reduced perfusion pressure in preterm infants and might explain why ventilation with 100% oxygen resulted in ~15% reduction in CBF in premature infants (40, 41). Exposure to supplemental oxygen at birth also resulted in prolonged cerebral vasoconstriction in preterm infants (6). Tsuji et al. (69) documented that a high oxygen saturation was associated with impaired cerebrovascular autoregulation and brain injury in premature infants. Niijima et al. (60) observed a fall in CBFV in healthy premature infants with hyperoxemia. Similarly, Leahy et al. (70) observed a significant decrease in CBF in healthy premature neonates after inhalation of 100% oxygen (25). Mechanisms of impaired autoregulation in hyperoxemia include oxidative stress, local production of vasodilators like nitric oxide, and direct vasoparalysis. These responses are gestational age dependent, which may explain that the data by Lundtrom et al. (45) in premature infants contrast to results in term equivalent animals that 100% oxygen increases CBF.

The result by Presti et al. (41) in mice is consistent with the report by Solas et al. (4) who demonstrated that 100% oxygen restored cerebral cortical microcirculation faster than 21% oxygen in piglets following hypoxia-ischemia (20). Even though threshold values for regional CBF after asphyxia have not been established, Solas et al. (39) concluded that a fast restoration of the cerebral microcirculation is beneficial as it was associated with less excitatory amino acids in the striatum. However, Presti et al. (41) demonstrated a trend toward a higher mortality among 100% resuscitated mice vs. mice resuscitated with 21% oxygen and concluded that 100% oxygen may be deleterious at the early stage of recovery due to reactive vasodilatation (71).

Limitations of this review include that we failed in optimizing our search strategy to capture studies that addressed our research questions. Thus, selection of the included studies was more subjective. We did not identify clinical studies that fulfilled our inclusion criteria, and studies in mainly severely asphyxiated post-transitional animals, as well as one study in premature infants were included. During perinatal transition with fluid filled lungs and less surface area for gas exchange, the effects of any given FiO_2_ is likely to differ from the post-transitional state. During initial stabilization, guidelines recommend that oxygen should be titrated to achieve a preductal SpO_2_ that is reflective of what healthy term infants experience. We identified no study that investigated weaning/titration of FiO_2_ after CC with 100% oxygen. None of the included studies reported PaO_2_ levels during CC. Most studies used SpO_2_ to measures blood oxygenation. SpO_2_ does not necessarily represent oxygen uptake and usage by organs, including the brain. Increased blood flow and/or oxygen extraction may serve to maintain oxygen delivery during hypoxemia. Thus, SpO_2_ alone provides limited information on oxygen consumption by tissues at high or low blood oxygen levels. Near-infrared spectroscopy (NIRS) measures tissue oxygen saturation continuously in a non-invasive manner (72), and was used in some of the studies. Finally, the use of CA flow as a surrogate of CBF could be criticized. However, Gratton et al. (73) demonstrated a direct correlation between CA flow and CBF during hypoxia and reoxygenation in lambs.

In conclusion, CPR with 100% oxygen may more rapidly restore CBF after hypoxia-ischemia, and increases cerebral oxygen delivery. Indeed, the latter may incur oxidative stress-related damage to the ischemic brain while systemic circulatory recovery is the same whether 21 or 100% oxygen is used during neonatal CPR. The advantage of using pure oxygen in neonatal CPR remains to be determined.

## Author Contributions

AS, GS, and P-YC: conception and design, collection, assembly, analysis, and interpretation of the data, drafting of the article, critical revision of the article for important intellectual content, and final approval of the article.

### Conflict of Interest

The authors declare that the research was conducted in the absence of any commercial or financial relationships that could be construed as a potential conflict of interest.
